# A Biological Model for Influenza Transmission: Pandemic Planning Implications of Asymptomatic Infection and Immunity

**DOI:** 10.1371/journal.pone.0001220

**Published:** 2007-11-28

**Authors:** John D. Mathews, Christopher T. McCaw, Jodie McVernon, Emma S. McBryde, James M. McCaw

**Affiliations:** 1 Vaccine & Immunisation Research Group, Murdoch Childrens Research Institute and School of Population Health, The University of Melbourne, Parkville, Victoria, Australia; 2 Centre for Clinical Research Excellence in Infectious Diseases, Victorian Infectious Diseases Service, The Royal Melbourne Hospital, Parkville, Victoria, Australia; 3 Department of Medicine, The University of Melbourne, Parkville, Victoria, Australia; University of Nottingham, United Kingdom

## Abstract

**Background:**

The clinical attack rate of influenza is influenced by prior immunity and mixing patterns in the host population, and also by the proportion of infections that are asymptomatic. This complexity makes it difficult to directly estimate *R_0_* from the attack rate, contributing to uncertainty in epidemiological models to guide pandemic planning. We have modelled multiple wave outbreaks of influenza from different populations to allow for changing immunity and asymptomatic infection and to make inferences about *R_0_*.

**Data and Methods:**

On the island of Tristan da Cunha (TdC), 96% of residents reported illness during an H3N2 outbreak in 1971, compared with only 25% of RAF personnel in military camps during the 1918 H1N1 pandemic. Monte Carlo Markov Chain (MCMC) methods were used to estimate model parameter distributions.

**Findings:**

We estimated that most islanders on TdC were non-immune (susceptible) before the first wave, and that almost all exposures of susceptible persons caused symptoms. The median *R_0_* of 6.4 (95% credibility interval 3.7–10.7) implied that most islanders were exposed twice, although only a minority became ill in the second wave because of temporary protection following the first wave. In contrast, only 51% of RAF personnel were susceptible before the first wave, and only 38% of exposed susceptibles reported symptoms. *R_0_* in this population was also lower [2.9 (2.3–4.3)], suggesting reduced viral transmission in a partially immune population.

**Interpretation:**

Our model implies that the RAF population was partially protected before the summer pandemic wave of 1918, arguably because of prior exposure to interpandemic influenza. Without such protection, each symptomatic case of influenza would transmit to between 2 and 10 new cases, with incidence initially doubling every 1–2 days. Containment of a novel virus could be more difficult than hitherto supposed.

## Introduction

Reports of past influenza pandemics show marked variation in clinical attack rates between populations. In the 1918–19 H1N1 pandemic, rates of clinical illness were less than 20% in some urbanised communities, but more than 60% in isolated communities such as Western Samoa [1]–[4]. During the 1968 H3N2 pandemic, attack rates in US households were limited to 30–40% [5], whereas almost the whole population fell ill when the virus reached the isolated island of Tristan da Cunha (TdC) in 1971 [6]. Biologically-based models for pandemic influenza [1] that incorporate effects of host immunity can help to explain such differences in observed attack rates. Such models could also explain recurrent waves of infection reported from 1918–19 [2]–[4] and 1968–71[6]. Higher attack rates in isolated populations are most likely due to fewer past exposures and lesser immune protection, leading to greater susceptibility. Multiple waves could reflect rapid waning of immune protection following exposure to a novel virus, antigenic drift [1], [4], seasonal influences on transmission of respiratory agents [7] or effects of social interventions [8].

Our flexible model, which we here apply to outbreaks of H3N2 from 1968–71 [6] and H1N1 from 1918 [2], allows for the possibility of asymptomatic infection [4], [9]–[11], for pre-existing immunity [4], [9], [10] and for the waning of immune protection and/or antigenic drift over time [1], [4], [10]–[12]. As the immune response cumulates following repeated exposure to seasonal variants of influenza [4], [12], and wanes thereafter, protection tends to be stronger in populations with a history of more recent exposure [4]. Our results also suggest that pre-existing immunity, arguably induced by prior exposure to inter-pandemic influenza, provided short-lived protection against the new pandemic virus of 1918. Such cross-reactive (heterosubtypic) immunity [4], [12]–[14] could also be important in protecting global populations against H5N1 or any other pandemic virus that might emerge.

## Methods

### Approach

Epidemic curves from single wave outbreaks with low rates of symptomatic influenza provide little or no information to separate the effects of viral exposure (and hence magnitude of *R_0_*) from the effects of population immunity or asymptomatic infection. Any arbitrary outbreak can arguably be explained by either high intensity exposure (high *R_0_*) with a high level of prior immunity or a high rate of asymptomatic infection, or alternatively by a low *R_0_* with less immunity, or by appropriate intermediate combinations [1]. Furthermore, measurement of subtype-specific antibody, as in a number of household and challenge studies [5], [10], [15] does not provide information about heterosubtypic immune protection induced by prior exposure to other subtypes [10], [13]. Multiple-wave outbreaks can provide more information about the interaction between influenza and the host immune system [1], particularly if there is evidence of repeated attacks in some individuals (See Appendix S1). Furthermore, as asymptomatic infection can induce immunity [9], [10], the time course of an outbreak also provides some information about asymptomatic infection. Accordingly, we have used data on the incidence of symptomatic influenza in two multiple-wave outbreaks of pandemic influenza, on different time-scales, to make inferences about asymptomatic infection, pre-existing levels of immunity, and the rate of change of immunity following exposure to a new pandemic strain. We chose not to use mortality or hospital admissions data because of the additional uncertainties arising from changes in virulence and incomplete reporting.

### Sources of data

The population of Tristan da Cunha, a remote island in the South Atlantic, had been free of influenza for 8–9 years when H3N2 was introduced by ship from South Africa in 1971[6]. The resulting epidemic curve over 50 days was based on reports of cases by day of symptom-onset. In two waves, 96% of the population of 284 fell ill; there were 365 recorded attacks, of which 312 could be identified with a precise day of onset. 273/284 islanders experienced a first attack of influenza, and 92/284 experienced a second attack. Most second attacks coincided with the second population wave; a minority of individuals experienced a first (and only) clinical attack during the second wave, possibly following asymptomatic infection during the first wave. Second attacks were generally less severe. Two elderly persons died [6]. To test the flexibility of the model to evaluate multiple wave behaviour over a longer time-frame, we also examined weekly reports of new cases of influenza among 180,000 personnel in RAF camps, providing apparently unbiased incidence rates for symptomatic influenza over 32 weeks of the summer and autumn waves of the 1918–19 UK pandemic [2]; data for the third (winter) wave were unavailable because of post-war demobilisation. The cumulative incidence of clinical illness was only 25% over the two waves of illness, arguably because some RAF personnel in the UK were protected because of prior seasonal exposure to interpandemic influenza.

### Basic epidemic model

**Figure 1:**
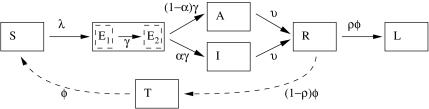
Flexible Influenza Model. Each compartment corresponds to a class of individuals in the population, and the arrows indicate the flows of individuals from class to class over time. As a result of exposure to the force of infection (*λ*), susceptible individuals (*S*) flow through two exposed classes of latent infection (*E1* and *E2*). A proportion (*α*) then become infective and symptomatic (*I*), and a proportion 1-*α* become asymptomatic (*A*). Both *I* and *A* pass to the recovered class (*R*) from which a proportion *ρ* develop longer-lasting protection (L), while the remainder eventually return to the susceptible class after passing through a temporary state (*T*).

Fig. 1 shows our extended *SEIRS* model [16]. After exposure to influenza virus, susceptible hosts (*S*) pass through two sequential exposed states (*E1* and *E2*) of latent infection, providing flexibility in the distribution of the estimated time spent in the infective and symptomatic state (*I*) that follows. The model also allows for individuals with asymptomatic or unreported infection (*A*). A key model assumption is that these two types of infections (*A* and *I*) occur in proportion, which ensures that model behaviour is independent of the degree of infectiousness of asymptomatic (*A*) cases. For mathematical simplicity, we may therefore consider the infectivity of asymptomatic cases to be zero (See Appendix S1). (However, as *A* cases become immune without being directly observed, the shape of the incidence curve does provide information about the frequency of *A* infections.) The recovered state (*R*) follows viral clearance from both *I* and *A* states. Recovery is followed by longer-term immunity (*L*) with formation or consolidation of ‘memory’ immune responses in a proportion, or by a temporary state of protection or immunity (*T*) in the more immunologically naive, before subjects again become susceptible (*S*) to re-infection. The full parameter set and the relevant differential equations for the model are described in Appendix S1. To explain the results in this main paper, we define:

*R_0_* = average number of secondary cases (*I* and *A*) from each infectious case (*I* and *A*) if all contacts are susceptible. If asymptomatic infections do not transmit, it is easy to see that *R_0_* is the average number of secondary symptomatic and infectious cases from each primary symptomatic and infectious case.

*λ* = force of infection (See Appendix S1);

*z* = proportion of individuals susceptible (*S*) prior to the first wave;

*α* = proportion of latent infections (*E2*) becoming infective and symptomatic (*I*);

1–*α* = proportion of infections that are asymptomatic or unreported (*A*);

2/*γ* = *T_e_* = mean time in states of latent infection (*E1* or *E2*);

2/*φ* = *T_w_* = mean time in resistant states (*R* or *T*);

1/*υ* = *T_i_* = mean time as *I* (infective) if symptomatic or as *A* if asymptomatic;

*ρ* = proportion of resistant (*R*) developing longer-term resistance (*L*);

### Serial interval, doubling time and transmissions per day

The mean generation time or serial interval for our SEIR model is *G* = *T_e_*+*T_i_*. The effective reproduction rate, *R_e_*, is defined as *z.R_0_*, where *z* is the proportion initially susceptible. The initial doubling time (*D*) is related to *R_0_* by the formula given in Roberts & Heesterbeek [17]. The mean number of transmissions per day for each transmitter in a fully susceptible population is estimated as *R_0_/T_i_*.

### Model-fitting

We used MATLAB v7.3 to fit deterministic epidemic curves and for Monte Carlo Markov Chain (MCMC) simulations to estimate parameter distributions; we used the negative binomial distribution to calculate each likelihood [18]. For TdC we fitted a prior distribution for the latent period (normal distribution with a mean of 1.25 days and variance of (0.3)^2^ days) and serial interval (log normal distribution with a mode of 2.6 days and variance of (0.33)^2^ days), based on estimates from the literature [1], [19]–[22]. We also used stochastic simulation, conditional on deterministic parameters, to fit the Tristan da Cunha observations, and construct empirical likelihoods. For the RAF simulations we fixed both *T_i_* and *T_e_*. More details are provided in Appendix S1.

## Results

### Epidemic curve for Tristan da Cunha in 1971

**Figure 2:**
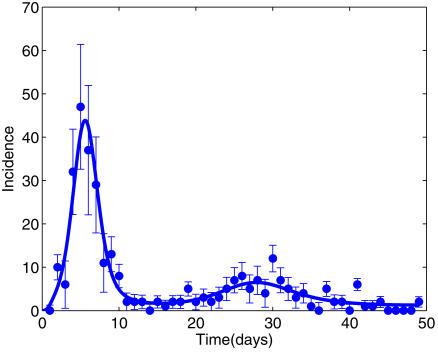
Incidence data and fitted model for Tristan da Cunha. Observed and fitted (median parameters) incidences for the H3N2 outbreak on the island of Tristan da Cunha in 1971 (cases per day, starting from 15^th^ August). Error bars (+/− one SD) are calculated using the negative binomial variance (See Appendix S1).

Fig. 2 shows the model fit and Table 1 summarises the corresponding parameter estimates and credibility intervals. The results indicate that about 84% of islanders were susceptible before the outbreak began (*z* = 0.84, 95% credibility interval 0.62–0.99)), as would be expected in a population that had been free of influenza for 8–9 years. 96% of persons reported clinical influenza in one or both waves [6]. Our model estimated that most islanders were exposed twice and that almost all infections led to clinical symptoms (*α* = 0.91, (0.72, 1.00)). Inferred exposures in the first wave on TdC led to protection of very short duration (median 12 days), allowing the second wave to cause (milder) second infections in some of those previously affected [6]. The estimated median infective period was 0.98 days (0.30, 1.83), and *R_0_* found support in the range 3.73–10.69 (Table 1).

**Table 1:** Parameters and summary statistics to explain influenza outbreaks with multiple waves.

Estimated quantity	RAF camps (1918)*	Tristan da Cunha (1971)*
*R_0_* = average number of secondary cases from each primary case in a fully susceptible population	2.88 (2.26, 4.28)	6.44 (3.73, 10.69)
*z* = proportion susceptible before first wave	0.51 (0.34, 0.65)	0.84 (0.62, 0.99)
*α* = proportion of people with latent infections who develop symptoms	0.38 (0.28, 0.60)	0.91 (0.72, 1.00)
*ρ* = proportion of infections followed by longer-lasting protection	0.55 (0.41, 0.70)	0.49 (0.39, 0.57)
2/*φ* = *T_w_* = mean time (days) in temporarily resistant state after infection	68 (56, 95)	12 (9, 17)
2/*γ* = *T_e_* = mean latent period (days)	1.30 (fixed)	1.36 (0.82, 1.87)
1/*υ* = *T_i_* = mean infective period (days)	1.00 (fixed)	0.98 (0.30, 1.83)
2/*γ*+1/*υ* = mean serial interval (days)	2.30 (fixed)	2.34 (1.56, 3.26)
Initial doubling time in fully susceptible population (days)	1.25 (0.85, 1.69)	0.62 (0.52, 0.73)
Initial doubling time in actual population (days)	3.93 (3.69, 4.19)	0.72 (0.63, 0.81)
Initial transmissions per day per transmitter in fully susceptible population	2.88 (2.26, 4.28)	6.76 (3.84, 16.35)
Initial transmissions per day per transmitter in actual population	1.46 (1.43, 1.50)	5.59 (3.20, 13.33)

* The 1918 pandemic is known to have been caused by H1N1; the 1971 outbreak on Tristan da Cunha was caused by H3N2.

Parameter values (median, 95% credibility intervals) were estimated by MCMC simulation (See Appendix S1). The estimate for the mean serial interval, the mean doubling times and transmissions per day per transmitter were derived from the full MCMC distributions.

### Epidemic curve for RAF Camps in 1918

**Figure 3:**
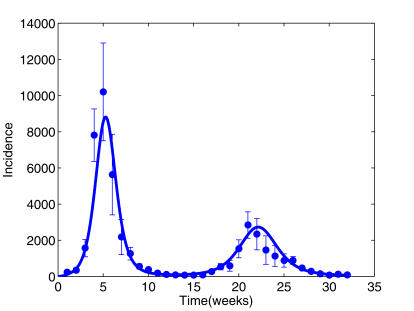
Incidence data and fitted model for RAF. Observed and fitted (median parameters) incidences of influenza reported from RAF camps in UK during the 1918 pandemic of H1N1 (cases per week, starting from week of June 8). Error bars (+/− one SD) are calculated using the negative binomial variance.

Due to the weekly reporting of influenza in RAF camps, our MCMC algorithm was unable to distinguish between a range of possible solutions, leading to wide credibility intervals on the estimates for most parameters (See Appendix S1). For this reason, we fixed the latent period (*T_e_*) and mean infectious period (*T_i_*) to the posterior median estimates from the TdC run. Fig. 3 shows the model fitted to the summer and autumn waves with *T_e_* = 1.3 days and *T_i_* = 1.0 days. Table 1 summarises relevant parameter estimates. The results indicate that only some 51% of RAF personnel were susceptible prior to the first wave (*z* = 0.51 (0.34, 0.65)), arguably because of recent exposures to inter-pandemic influenza. Only 38% of exposures of susceptible persons led to clinical symptoms (*α* = 0.38 (0.28, 0.60)). In this case, inferred exposures led to temporary protection of about 68 (56, 95) days duration, thus accounting for the longer delay between the summer and autumn waves (Fig. 3). Over the two waves, the cumulative incidence of symptomatic attacks for RAF personnel was only 24.7%. *R_0_* found support in the range 2.26–4.28. The assumption that all RAF personnel were initially susceptible led to a significantly worse model fit, and if all infections were additionally assumed to be symptomatic, the model was unable to adequately reproduce observed epidemic behaviour (See Appendix S1).

### Estimates of doubling time

Initial doubling times for influenza were estimated as 0.72 days on Tristan da Cunha and 3.93 days in RAF camps. In fully susceptible populations, the estimated doubling times would be 0.62 days and 1.25 days respectively.

### Transmissions per day

For Tristan da Cunha, there were initially 5.59 (3.20, 13.33) transmissions per day for each transmitter, corresponding to 6.76 (3.84, 16.35) if the population were fully susceptible. For RAF personnel, the estimates were 1.46 (1.43, 1.50) and 2.88 (2.26, 4.28) per day respectively.

## Discussion

Our flexible model explains multiple waves of influenza by incorporating biological effects that have been overlooked in some earlier pandemic models. The model allows for the possibility that asymptomatic infection [4], [9], [10], pre-existing immunity, and waning immunity or antigenic drift of the virus [4], [9], [10], [12] could affect transmission and disease, However, it should be emphasised that the magnitudes of each effect in the fitted model were determined by the data. Where an effect was missing or small, as with the low proportion of Tristan da Cunha islanders with prior immunity, this could be inferred from the parameter estimate (Table 1).

Seasonality [7] and social distancing measures [8], [22], [23] might also explain multiple waves of influenza. However, it is unlikely that seasonality alone can explain multiple waves occurring over weeks to months in the absence of waning immunity, antigenic drift and/or birth of new susceptibles. At least for seasonal interpandemic influenza, seasonal forcing seems to determine the *timing* of a new outbreak, whereas the *magnitude* is likely determined by *R_0_* and the proportion susceptible. Furthermore, as the first wave of pandemic flu in 1918 in the UK was in summer and out of season, it must have been triggered by “non-seasonal” factors (i.e. new virus in a (partially) susceptible population). We cannot exclude an effect of season on the timing of the second wave in 1918, nor can we exclude a role for social interventions in explaining the gap between the first and second waves in 1918. However, our inference that waning immunity can replenish the susceptible pool over time scales of months (RAF) or weeks (TdC) is biologically plausible and arguably more parsimonious.

Asymptomatic influenza infections are known to be immunising [9], [10], which helps to explain why the clinical attack-rate does not approach 100%, even in isolated, immunologically naïve populations, as in Samoa and Alaska in 1918–19 [2]–[4], where expected *R_0_* would have been high. We estimated that about 59% of infections of susceptible RAF personnel in 1918 were asymptomatic, compared with only 9% on TdC in 1971. This difference suggests that the clinical attack rate was reduced in RAF personnel by two mechanisms–firstly by a lower proportion of susceptibles, and secondly by the higher proportion of infections in susceptible persons that were asymptomatic or unreported. High rates of asymptomatic infection [9], [10] with the capacity for transmission can also help to explain why chains of influenza transmission are often unidentifiable in inter-pandemic years, particularly in urban settings [4], [9], [11].

Our results suggest that prior immunity was important in protecting against clinical attack in the 1918 H1N1 pandemic, but do not explain the origins of that immunity. However, heterosubtypic immunity likely provides at least some protection against influenza A of novel subtype [4], [12]–[14], [24], [25] and specific antibodies against a new subtype can develop even when the inducing infection is asymptomatic [9]. We suggest that residual heterosubtypic immunity from the previously circulating H2 or H3 (interpandemic) viruses [4], [26] might account for the apparent lack of susceptibility in many RAF personnel before the summer wave. However, we cannot exclude the alternative possibility that the H1N1 virus might have circulated in less virulent form in the spring of 1918, as was suggested for the USA [27], [28], and thus immunized some individuals against later infection. Nevertheless, the three waves seen in 1918–19, and our detailed model for the first and second waves, suggest that pre-existing immunity was often short-lived, as was the immunity induced by first exposure to the novel virus. Unfortunately, we have no evidence that would allow us to separate the effects of waning immunity from the effects of antigenic drift of the 1918 pandemic virus.

One result from our Tristan da Cunha model could seem counter-intuitive: exposure in the first wave did not always protect against re-infection in the second wave several weeks later, and protection apparently waned much more quickly than in the RAF population (Fig. 2 and Table 1). Re-infections over similarly short time-intervals have also been described in an institutional population of young and susceptible naval apprentices [1], [29] and in other historical sources [2]. We have suggested [1] that initial viral clearance, involving innate immunity and cytokines [30], is not immediately followed by acquired immunity, especially in persons with little recent experience of influenza, as on TdC, where immune priming for influenza could have been absent or immune memory lost.

Our analyses have provided an economical explanation for the time course of the observed data in two contrasting outbreaks. Rather than providing inconsistent evidence, we suggest that the two outbreaks provide complementary evidence about how “immunity” to influenza can evolve over different time scales from different starting points. The dynamics of multiple-wave outbreaks on these different time-scales are at least partly due to the past exposure history of the population. We did not expect, and did not observe, comparable estimates for the waning time of immunity in the RAF and TdC populations.

Our inferred values of 2–10 for *R_0_* are consistent with some reports [15], [Bibr pone.0001220-Roberts2] [Bibr pone.0001220-Fraser1] [32] but are greater than some estimates used for pandemic planning [20], [33]–[35]. The marked difference in *R_0_* between TdC and RAF populations (Table 1) is unlikely to be due to differences between the H3N2 virus in 1971 and the H1N1 virus in 1918, because in an isolated UK boarding school population, we found that the 1918 virus spread with an *R_0_* of 6.90 (See Appendix S1). We suggest therefore that the *R_0_* difference is partly determined by differences in levels of prior immunity, arguably through reduced levels of viral shedding from persons with partial immunity. Differences in social mixing or stratification of the RAF population between different camps could also contribute to a lower *R_0_*. We note that after the arrival of the ship from South Africa there were welcome-home parties on TdC that could have contributed to the explosive outbreak over the first few generations of infection (6).

For the RAF outbreaks, with data reported only at weekly time steps, there is little information to allow MCMC estimation to separate the effects of changing serial interval from the effects of changing *R_0_*; likewise it was difficult to make inferences about the relative contributions of *T_e_* and *T_i_* to the serial interval. Table 1 provides parameter estimates and credibility intervals for RAF analyses where *T_e_* and *T_i_* were fixed. RAF results were similar, although less stable when *T_e_* and *T_i_* were constrained only by the priors on latent period and serial interval (See Appendix S1). For TdC, with data reported daily, information on the latent period can be extracted by the model, which is able to resolve the subtle timing differences that arise from trading off *T_e_* and *T_i_*. Pleasingly, our posterior median latent period was consistent with our prior at 1.36 days.

The estimates for the latent period from TdC simulations (See Appendix S1) are close to published estimates of mean incubation period (time from exposure to onset of symptoms) of 1.48 [21] or 1.9 days [20], leaving a short time window before the onset of symptoms during which a person could be infective for others. Our estimate of median infective period (1.01 days for TdC) is short compared with the duration of viral shedding [1], [30], and with some other estimates of mean infective period [22]. This discrepancy suggests that the process of infecting other people can be terminated by isolation of cases, or by exhaustion of susceptibles in the local environment, as well as by a decline in viral shedding. If substantial transmission occurs before the onset of symptoms, as suggested by others [31], this would add to the difficulties of controlling any new pandemic.

Our flexible model, with host immunity and asymptomatic immunising infections as the constraints on observable disease spread, adequately reproduces the observed epidemiology of influenza in disparate populations, and leads to additional insights into virus behaviour. Less flexible models can explain single wave outbreaks with a range of *R_0_* values, but have little capacity to estimate immune effects [1]. Indeed, several reports have implied that it is unimportant to distinguish between *R_0_* and effective *R_e_*, as it is the latter that largely determines the rate of population spread [15], [20], [34]. However our model uses the additional information from multiple wave outbreaks to draw stronger inferences about immunity and asymptomatic infection, as well as *R_0_* and mean infective period.

What might our findings mean for pandemic planning? The bad news is that the pandemic doubling time in a fully susceptible population could be as short as 1 or 2 days, and that *R_0_* for a pandemic strain could be considerably higher than has been assumed in some previous models [1]. However, the good news is that high levels of pre-existing immunity could translate a high *R_0_* into a much lower effective *R_e_*. Unfortunately, heterosubtypic immunity, which can be short-lived, is likely induced more effectively by recent infection with a live influenza virus than by conventional sub-unit vaccines [14], [24]. This raises the possibility that inter-pandemic sub-unit vaccine, by preventing infection with live inter-pandemic virus, could even make people more susceptible to a novel pandemic virus. The bottom line is that we need much more information about heterosubtypic immunity in humans, and about the potential value of live-attenuated influenza vaccines against H1N1 and H3N2 in protecting populations against H5N1 or any other novel pandemic virus. We await the results of relevant research with great interest.

## Supporting Information

Appendix S1 (0.50 MB PDF)
