# Supplementary material for: A Biological Model for Influenza Transmission: Pandemic Planning Implications of Asymptomatic Infection and Immunity
Source: PLoS One. 2007 Nov 28;2(11):e1220. doi: 10.1371/journal.pone.0001220 (PMC2080757; doi:10.1371/journal.pone.0001220)
Supplement: Appendix S1 — (0.50 MB PDF) [file pone.0001220.s001.pdf]

A biological model for influenza transmission. Pandemic planning implications of asymptomatic infection and immunity: Appendix S1

John D. Mathews*, Christopher T. McCaw*, Jodie McVernon*,
Emma S. McBryde† and James M. McCaw*‡

November 2, 2007

Contents

S1 Details of the model 2
S1.1 Model structure 2
S1.1.1 Asymptomatic infections and the definition of R_0 2
S1.1.2 Differential equations 3

S2 Model fitting 3
S2.1 Multiple wave data 3
S2.2 Markov Chain Monte Carlo 3
S2.3 Tristan da Cunha 4
S2.4 RAF 5

S3 Results 5
S3.1 Validity of posterior medians and credible intervals 5
S3.2 Details of the MCMC calculations for Tristan da Cunha and RAF 7
S3.3 Simpler models for RAF 7
S3.4 A stochastic model for the island of Tristan da Cunha 11
S3.5 Ancillary results from a boarding school in 1918 12

S4 Advantages and limitations of our model 13

References 13

*Vaccine & Immunisation Research Group, Murdoch Childrens Research Institute and School of Population Health, University of Melbourne, Australia
†Victorian Infectious Diseases Service, Royal Melbourne Hospital and Department of Medicine, University of Melbourne, Australia
‡Corresponding author. Electronic mail: jamesm@unimelb.edu.au, Phone: +61 3 8344 9145, Fax: +61 3 9348 1827.

Abstract

We provide details of the biologically plausible model used to derive estimates for R_0, prior immunity, asymptomatic infection and waning time. The Markov Chain Monte Carlo (MCMC) fitting algorithm is described. The posterior parameter estimates are presented. A stochastic model for Tristan da Cunha is presented for comparison. Advantages and limitations of the model and scope of applicability of the results is discussed.

S1 Details of the model

S1.1 Model structure

The compartmental diagram for the model is presented in Figure S1. The two stage process for the latent period, and also for the transition from R back to S gives both periods a peaked distribution. Because of the complexity of incremental immune response to influenza, the residence times in the R, T and L states will vary according to the prior experience of the population, the virus, and the length of follow up (in terms of data collection).

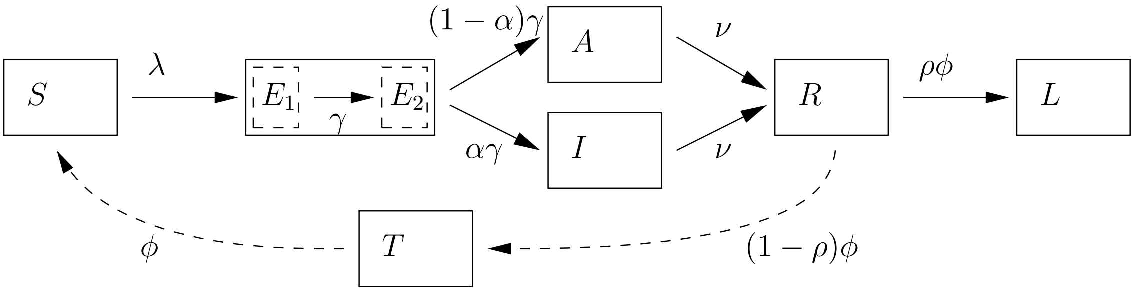

Figure S1: Individuals flow from the susceptible class (S) to a two-sub-state latent phase (E_1 and E_2). A proportion, α become symptomatic (I), while the remainder are asymptomatic (A). Once recovered (R), a proportion, ρ acquire longer term protection (L) while the remainder become susceptible once again (returning to S via the intermediate sub-state T.

S1.1.1 Asymptomatic infections and the definition of R_0

R_0 is the threshold condition for an epidemic in a fully susceptible population. In its most general form, it is the number of exposures (leading to both I and A cases) per transmitter (I or A).

In the absence of detailed information about who might have infected whom, the population incidence of reported symptoms provides no information on the infectiousness of asymptomatic transmitters. We therefore assume that a proportion, α, of all latent infections results in symptomatic illness (I). The remainder, a proportion $1 - \alpha$, are asymptomatic (A). Further, under the assumption that symptomatic and asymptomatic cases have the same duration of infection ($1/\nu$), the model is independent of the degree of infectiousness of asymptomatic cases as we have $A(t) = (1 - \alpha)/\alpha \times I(t)$.

As asymptomatic cases are removed from the susceptible population, they do affect the shape of the epidemic curve, thus allowing inferences to be made about α. The proportionality of A and I allows us to treat asymptomatic infections as if they were non-transmitting. We also note that if some symptomatic cases go unreported, on a random basis, then these will be implicitly added to those that are asymptomatic. Hence, the asymptomatic group might be more properly described as "asymptomatic or unreported".

S1.1.2 Differential equations

The force-of-infection is given by:

$$\lambda = \frac{R_0 \nu}{N\alpha} I. \tag{S1}$$

The coupled differential equations that require solving are:

$$\frac{dS}{dt} = -\lambda S + \phi T \tag{S2}$$
$$\frac{dE_1}{dt} = \lambda S - \gamma E_1 \tag{S3}$$
$$\frac{dE_2}{dt} = \gamma E_1 - \gamma E_2 \tag{S4}$$
$$\frac{dI}{dt} = \alpha\gamma E_2 - \nu I \tag{S5}$$
$$\frac{dA}{dt} = (1-\alpha)\,\gamma E_2 - \nu A \tag{S6}$$
$$\frac{dR}{dt} = \nu\,(I + A) - \phi R \tag{S7}$$
$$\frac{dT}{dt} = (1-\rho)\,\phi R - \phi T \tag{S8}$$
$$\frac{dL}{dt} = \rho\phi R \tag{S9}$$

subject to the initial conditions $S(0) = Nz$, $R(0) = N(1-z)$, $I(0) = I_0$ (data set dependent, see below) and $E_1(0) = E_2(0) = A(0) = T(0) = L(0) = 0$. N is the total number of individuals in the population for the outbreak in question, assumed fixed. z is the proportion initially susceptible. The effective initial reproduction number is given by $R_e = zR_0$.

S2 Model fitting

We used Markov Chain Monte Carlo (MCMC) techniques to fit our model to incidence data. We set the unit of time for Tristan da Cunha to one day. For RAF, we set the unit of time to one week. It follows that the incidence is best estimated from the model as the cumulative incidence over a unit of time:

$$\text{Incidence}(t) = \int_{t-1}^{t} \alpha\gamma E_2(\tau)\, d\tau. \tag{S10}$$

S2.1 Multiple wave data

Multiple wave outbreaks allow for more than just the waning rate to be estimated. The degeneracy between R_0 and the level of pre-existing immunity is at least partially broken, as evidenced by the stability of the estimates of R_0 and z for TdC.

S2.2 Markov Chain Monte Carlo

We used a standard MCMC algorithm to fit incidence data, $x(t)$, using a negative binomial variance with mean $m(t)$ determined by the current estimate and $r = 10$:

$$\Pr(x|m,r) = \binom{r+x-1}{x} \left(\frac{r}{r+m}\right)^r \left(\frac{m}{r+m}\right)^x. \tag{S11}$$

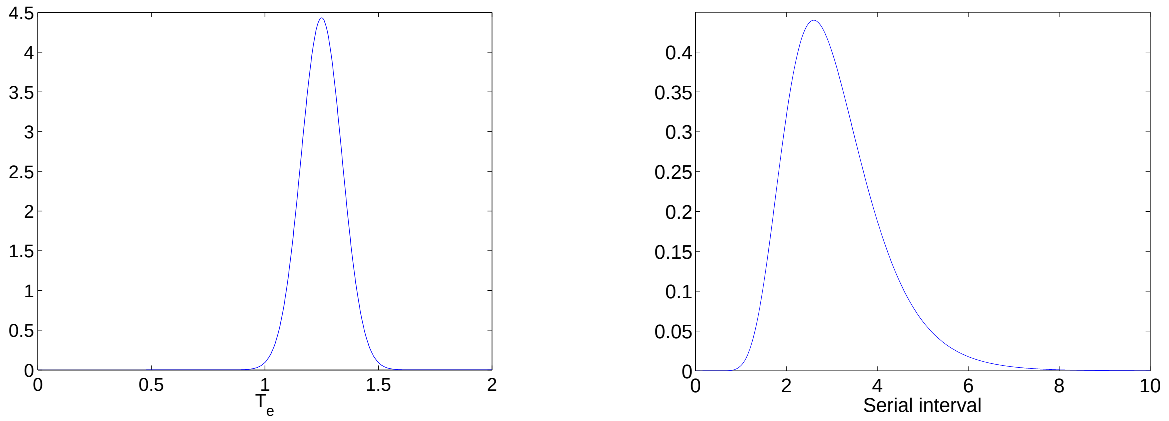

(a) (b)

Figure S2: **a.** Prior probability density function for latent period, T_e. **b.** Prior probability density function for the serial interval, $T_e + T_i$.

We used a flat prior for R_0, T_w, α, ρ and z. We thought it likely that the mean latent period ($T_e = 2/\gamma$) would be conserved, whereas the mean infective period ($T_i = 1/\nu$), influenced by population mixing as well as by viral characteristics, might vary with social circumstances.

Using data on viral shedding and incubation period we placed a normal prior on the average latent period T_e with a mean of 1.25 and variance of $(0.3)^2$. As T_i may differ across populations, we placed a prior not on T_i but rather on the serial interval, $T_e + T_i$. We used a lognormal distribution with mean 1.065 and variance $(0.33)^2$. The mode for the serial interval prior is approximately 2.6 days. Figure S2 shows the priors for T_e and the serial interval.

We ran chains for an adequate number of iterations to test for convergence of the MCMC process. In cases where the model was not initiated from within the posterior distribution, we discard an appropriate number of iterations as "burn in". For each parameter, we report the median and 95% credibility interval from the full distribution.

Although TdC simulations converged with the prior on latent period and serial interval, the RAF simulations did not converge, even after three million iterations. We attributed this lack of convergence to the fact that RAF data, recorded only at weekly intervals, could provide little or no information to discriminate between the contributions made by T_e and T_i to a serial interval of only a few days. When we fixed $T_e = 1.3$ and $T_i = 1.0$ for the RAF population, we did achieve convergence. The RAF estimates for the other parameters were consistent with (unconverged) estimates obtained with just the prior on latent period and serial interval, but with tighter credibility intervals.

To test the hypothesis that populations were fully susceptible, and/or all infections were symptomatic, we calculate the Bayesian Information Criterion (BIC) [1] for each MCMC model run. If the BIC improves by more than the number of new free parameters, we conclude that the more highly parameterised model is superior.

S2.3 Tristan da Cunha

Influenza was introduced to the island of Tristan da Cunha when the vessel *Tristania* arrived on 13th August 1971 carrying islanders returning from Cape Town. Two passengers displayed symptoms of acute respiratory disease immediately after landing. Accordingly, we set the initial seed (number of infectives) to $I_0 = 2$. We begin our deterministic model run from the beginning of the 15th August, although we obtain similar results if we run the model from the 14th August (results not shown). With

a model run beginning from the 15th August, we ignore the single infection recorded on the 14th. Similarly, the single infection recorded for October 10th (some seven days after the previous recorded infection) is dropped from the data set. Therefore, we have a total number of 310 recorded infections, rather than 312 as found in the original epidemic curve.

It is known that 365 symptomatic infections occurred over the two waves, but only 312 are known to within a single day's accuracy. In the absence of any specific information on when the remaining 53 were infected we assume they occurred in proportion to the recorded incidence. Therefore we must fit

$$\text{Incidence}(t) = g \times \int_{t-1}^{t} \alpha\gamma E_2(\tau)\, d\tau, \tag{S12}$$

where $g = 310/365$, allowing for the two cases dropped from the data set. Accounting for the known missing symptomatic cases in this way ensures that all infections entering the A box are truly "asymptomatic or unreported".

S2.4 RAF

The RAF data are recorded at weekly intervals, per 10,000 population. While the population size fluctuated somewhat over the course of the two waves, we assume a fixed population in our model runs of 180,000. We set $I_0 = 18$. Allowing I_0 to vary did not result in a significant improvement in fit (as determined by BIC) — in fact, after an MCMC run of $600,000$ iterations, the favoured estimate for I_0 was 19 (CI range 11–30). There were no reported cases where the week of onset was unknown and thus $g = 1$.

S3 Results

Here we show details of the MCMC model runs for Tristan da Cunha and RAF as well as introduce a stochastic model that has been used to validate results for the Tristan da Cunha population.

S3.1 Validity of posterior medians and credible intervals

With MCMC methods, the estimation process explores the parameter space to find the combinations of parameter values that could have generated the observed data. In our model, as is usual in MCMC, some of our recovered parameter estimates are highly correlated with each other. For example, as would be expected on theoretical grounds when there is only coarse timing data, estimates of z and R_0 have a strong negative correlation (see Figure S3). This correlation is less evident in the TdC simulations where there is sufficient information to estimate R_0 independently of z (see Figure S4). If the covariation between pairs of parameter estimates were approximately linear over the range of both estimates, then it would be reasonable to conclude that a model using the posterior median (or perhaps mean) for each parameter, would best predict the data. Unfortunately, the covariance relationship is non-linear for at least some pairs of parameters which means that prediction based on the total set of posterior medians or means is not necessarily optimal. In other words, although the posterior mean for each parameter indicates the single "best estimate" for that parameter, it does not tell us how good it is in combination with the posterior medians of the other parameters. Thus it is important to emphasise the credible intervals for each parameter and to not place undue emphasis on the posterior medians or means.

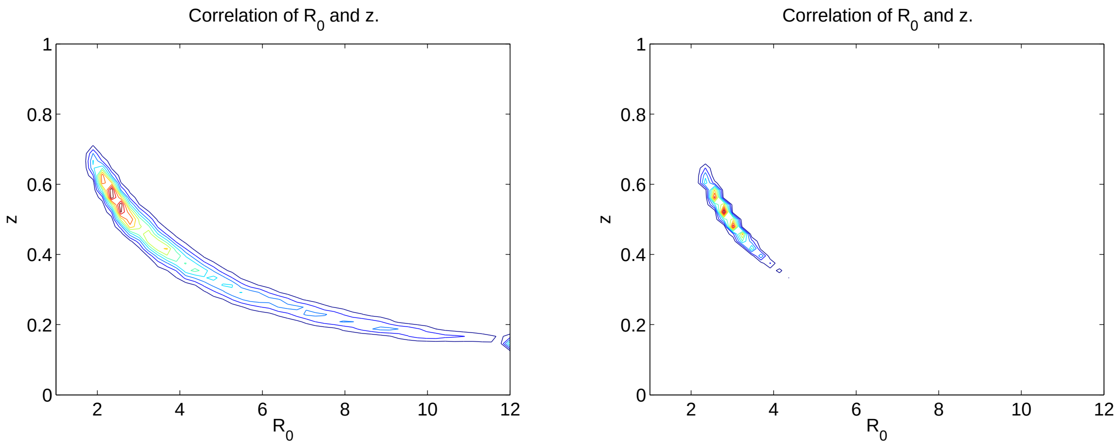

Figure S3: Correlation of z and R_0 for RAF. **a.** The seven parameter RAF fit. The correlation coefficient is -0.910. With a variable serial interval (latent plus infectious period) the model fit explores an overly wide range of parameter space. **b.** The five parameter RAF fit (T_e and T_i fixed). The correlation coefficient is -0.966. By fixing the infectious period (T_i) we constrain the feasible values of R_0 and hence z.

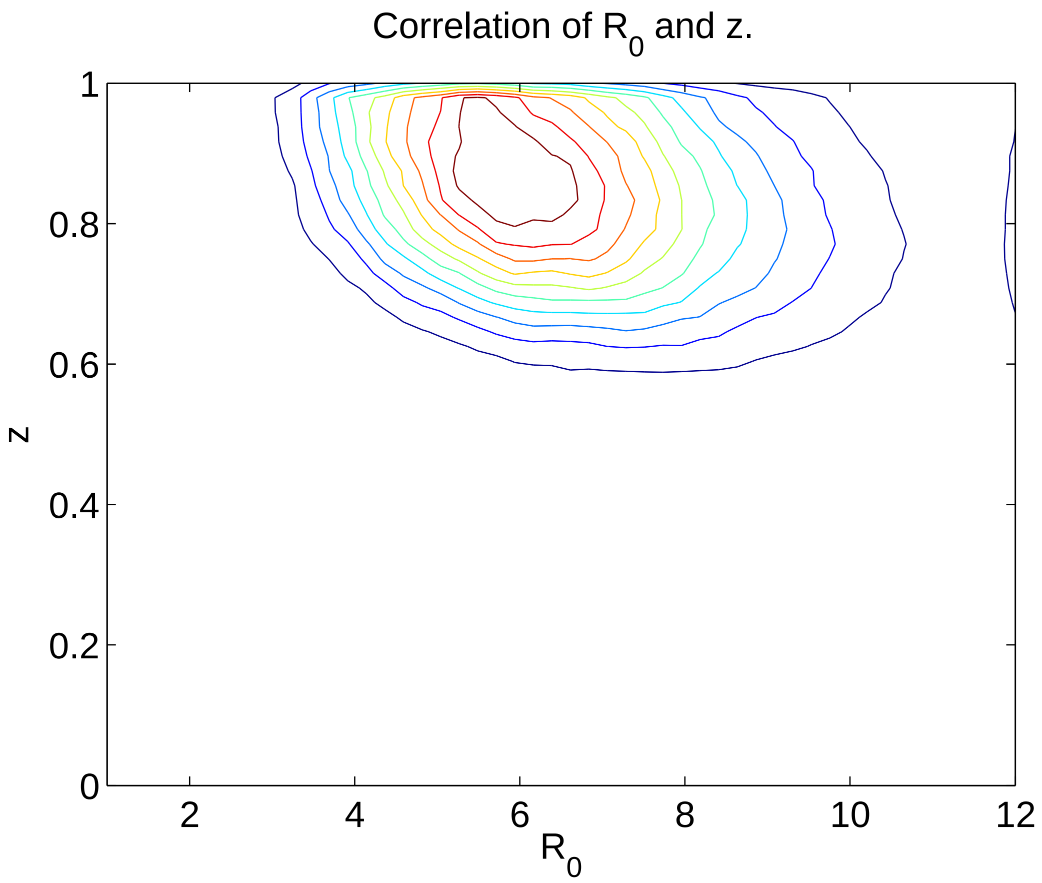

Figure S4: Correlation of z and R_0 for TdC. The correlation coefficient is -0.238. In contrast to RAF, the model is able to estimate z and R_0 independently.

S3.2 Details of the MCMC calculations for Tristan da Cunha and RAF

Figure S5 and Figure S6 show the MCMC chains and a histogram for each of the seven parameters for Tristan da Cunha and RAF respectively. Figure S7 shows the RAF run with the latent and infectious periods fixed at the posterior median values from the Tristan da Cunha run.

After three million iterations using TdC data, the posterior mean for the serial interval was 2.34 days comprising a mean latent period of 1.36 days and a mean infective period of 0.98 days.

From RAF data the posterior estimate of mean serial interval, after three million iterations, was 2.59 days comprising a mean latent period of 0.93 days and a mean infective period of 1.71 days. Inspection of the parameter distributions (Figure S6) for the RAF model shows some apparent instability, at least in part because incidence data measured at weekly intervals could provide little information to separate the effects of T_e and T_i on the serial interval of infection. How might this instability affect the credibility of the parameter estimates? The simulations show regular "spikes" where very high values of R_0 coincide with high value of T_w and α and low values of z. This simply means that those values can explain the data, but only in those particular combinations, which are not often seen. In other words, the credibility of that combination of parameter estimates needs to be judged on grounds of biological as well as statistical plausibility. In Figure S7, where T_e and T_i are fixed, the covariation of parameter estimates is reduced.

S3.3 Simpler models for RAF

For the RAF data fit (see main paper for results) the posterior median value for z, the proportion initially susceptible is 0.51. This value lies in stark contrast to the typical value assumed for a pandemic scenario of $z = 1$ (a fully susceptible population). To explore the validity of our conclusions, we have run our model with $z = 1$ fixed. The resulting best fit, presented in Figure S8a looks reasonable by eye. The R_0 value returned by the MCMC method is 1.60 (1.37 – 1.97). However, allowing for z to vary yields a significantly improved fit — the BIC improves from 476.5 to 426.1, a change of 50.4 for the additional complexity of one extra free parameter. It is also worth noting that, with a fully susceptible population, the inferred symptomatic proportion is extremely low ($\alpha = 0.18$ (0.15 – 0.24)). It is clear that including the possibility of prior immunity provides a more accurate explanation of the data.

Similarly, we can also disallow asymptomatic infection ($\alpha = 1$) when fitting the RAF data. With $z = 1$ still held, we find a best fit with $R_0 = 1.08$. The fit, however, is rather poor (Figure S8b). The BIC is 536.7, significantly greater than the BIC for both the full model and the model without prior immunity.

In summary, allowing for asymptomatic infection and prior immunity results in vastly improved fits to data. Furthermore, a model without such states, while in some cases capable of producing reasonable "by eye" fits, returns biologically implausible estimates for key parameters such as α (in the case $z = 1$) and R_0 (in the case $\alpha = z = 1$).

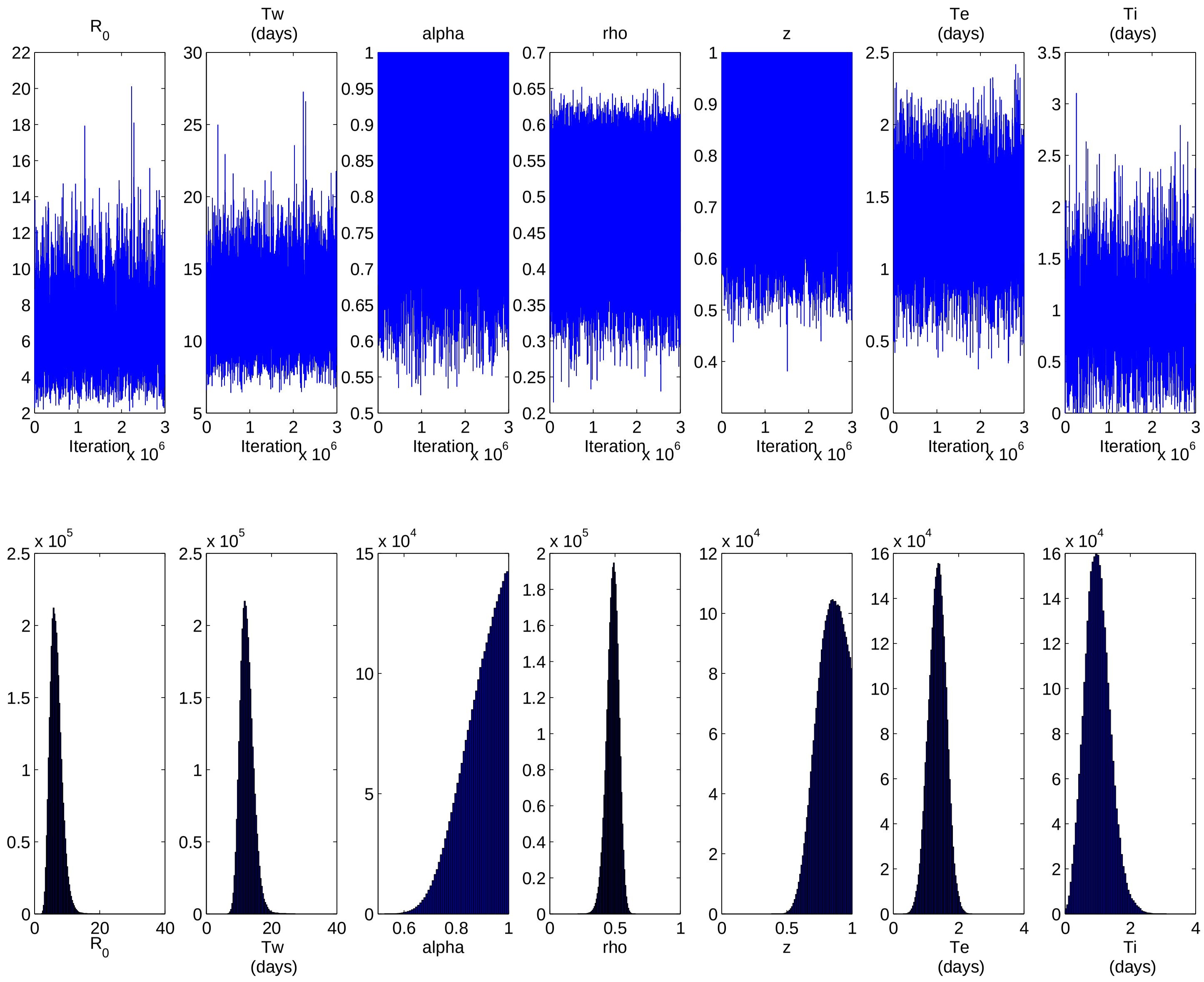

Figure S5: Parameter distributions by MCMC simulation for the Tristan da Cunha outbreak. Distributions are derived from one million simulations with negative binomial variance. $T_w = 2/\phi$ is the mean time in days for temporary protection to wane. $T_e = 2/\gamma$ is the mean latent period. $T_i = 1/\nu$ is the mean infective period.

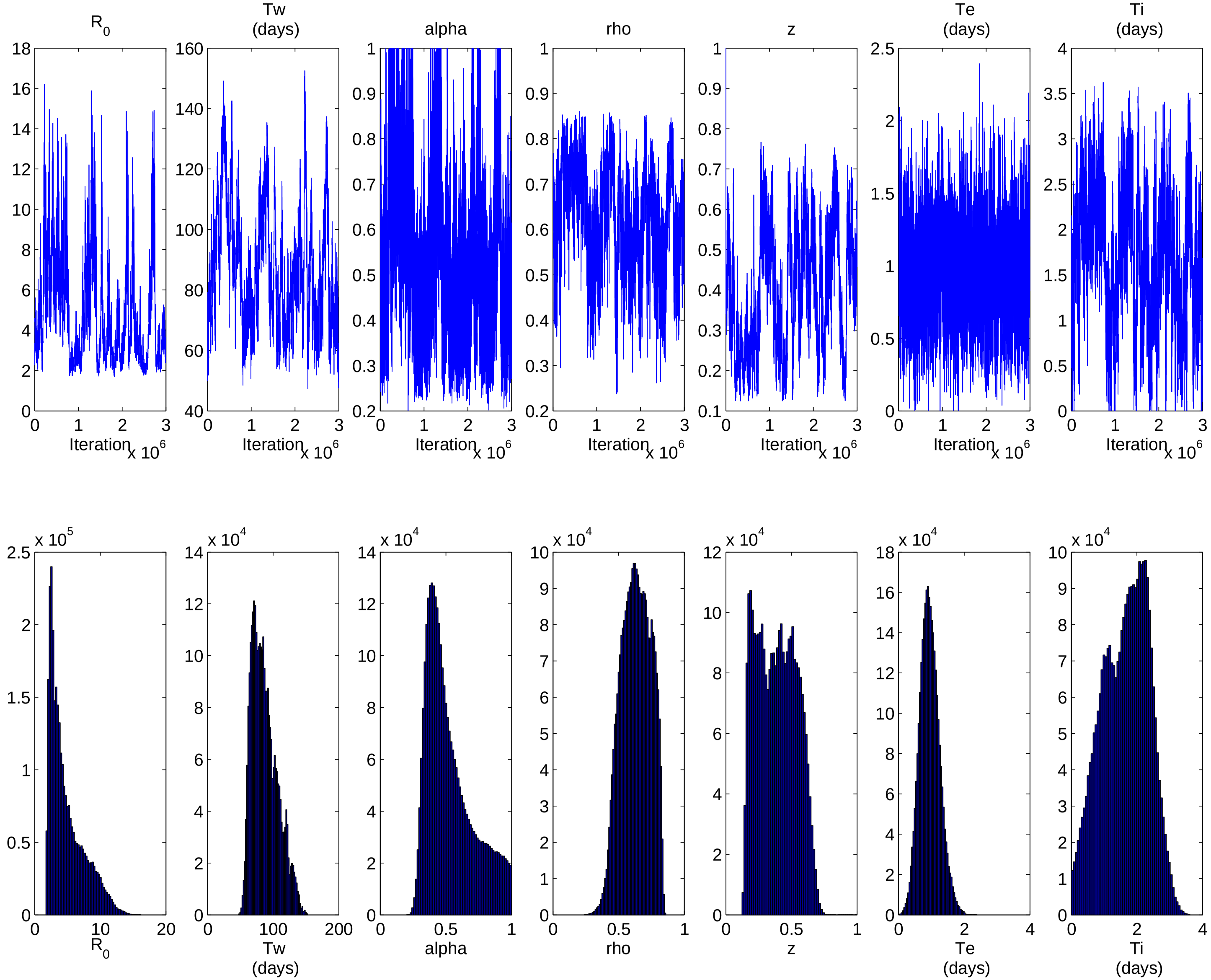

Figure S6: Parameter distributions by MCMC simulation for the RAF outbreak, derived from three million simulations. Details as in Figure S5

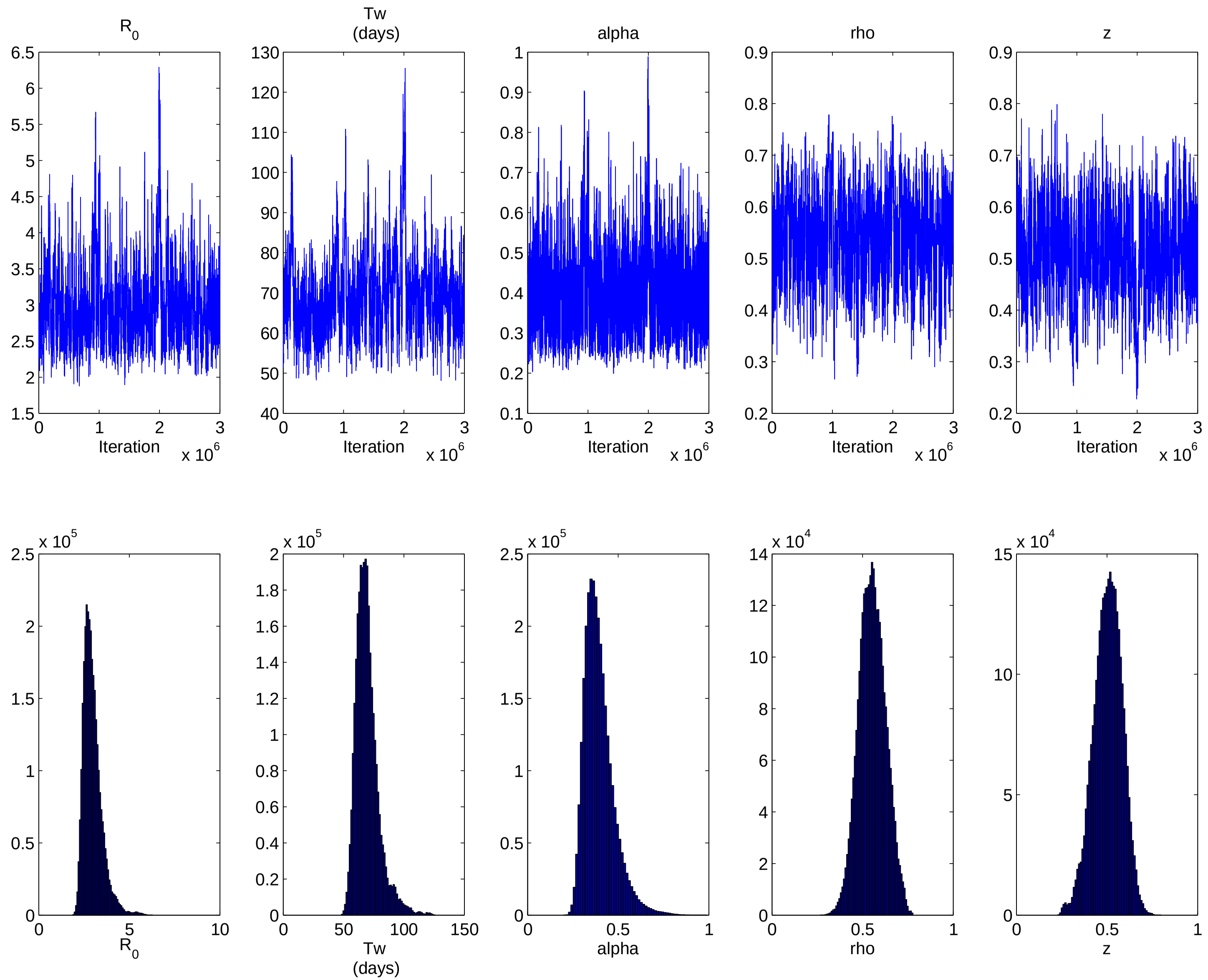

Figure S7: Parameter distributions by MCMC simulation for the RAF outbreak, derived from three million simulations, with $T_e = 1.30$ and $T_i = 1.00$.

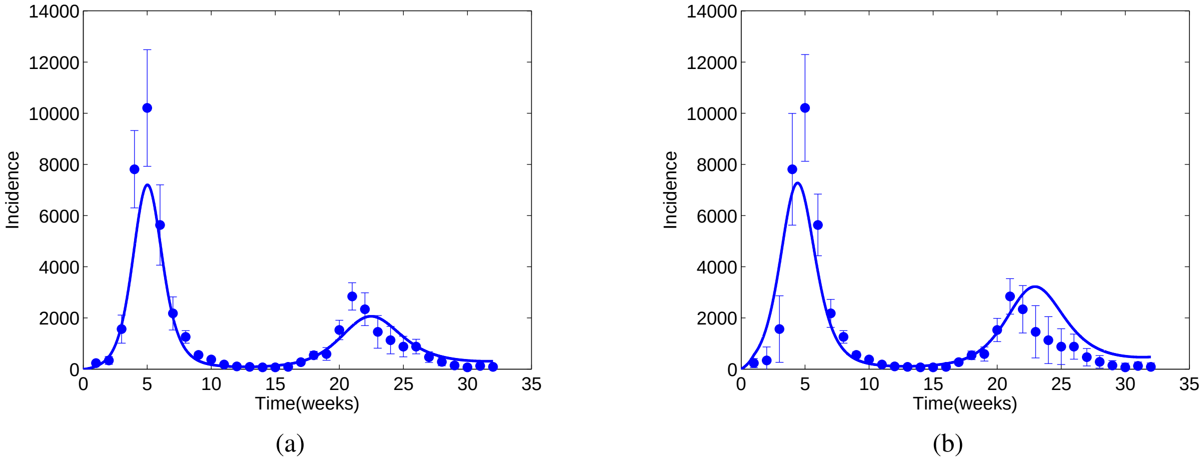

Figure S8: Epidemic curve (median parameters) for RAF, **a.** with $z = 1$ fixed (2 million runs, $R_0 = 1.60$, $\alpha = 0.18$, $\rho = 0.20$, $T_w = 52$, $T_e = 1.32$, $T_i = 1.26$); **b.** with $z = 1$ and $\alpha = 1$ fixed (1.5 million runs, $R_0 = 1.08$, $\rho = 0.05$, $T_w = 59$, $T_e = 0.06$, $T_i = 0.39$).

S3.4 A stochastic model for the island of Tristan da Cunha

The small population size on Tristan da Cunha ($N = 284$) means that there is a significant chance of extinction at the beginning of the outbreak and between the two waves. Here we present results from a simple stochastic model of infection for the outbreak on Tristan da Cunha.

We use the deterministic model shown in Figure S1 to calculate the transition rates between states. We assume a poisson distribution, conditioned on the current state, for these rates. The model is calculated in continuous time. Only one event occurs at any time and the model is updated after each event. Rather than examine the range of possible outcomes given our parameter values, we choose to examine the question: "How likely is it that the stochastic process resulted in the observed epidemic?" We do so by conditioning on the observed data after each unit of time (one day). We have seven hidden states in the model (S, E, I, A, R, T and L) and observe only the incidence on each day.

For each day n, we record how many stochastic runs are required, starting from day $n-1$, to obtain the observed incidence. If we are unable to match the observed incidence after a certain number of stochastic runs, we step back to day $n - 2$ and recalculate the number of runs required to match at day $n - 1$, appending this number of new runs to the existing run number for day $n - 2$. Due to the stochastic nature of the calculation, upon matching for day $n - 1$ we will have a different set of hidden states and thus may successfully match for day n. We calculate an empirical log-likelihood as the sum of the negative log of the number of attempts required to match each data point:

$$LL = -\sum_{n=1}^{49} \log\left(1 + x_n\right), \tag{S13}$$

where x_n is the number of trials required to match for day n. A full run is deemed successful if the stepping algorithm succeeds in negotiating from $t = 0$ (15th August) through to the end of the epidemic (2nd October) (49 data points).

At each day in the stochastic simulation, we check for extinction (conservatively defined when all of E_1, E_2 and I are zero). Extinctions are treated in the same way as failed matches to data — the model steps back one (or more) days. An example run from the stochastic model using the posterior median MCMC parameters is shown in Figure S9a. Figure S9b shows a histogram of the empirical negative-log-likelihoods over $10,000$ stochastic model runs.

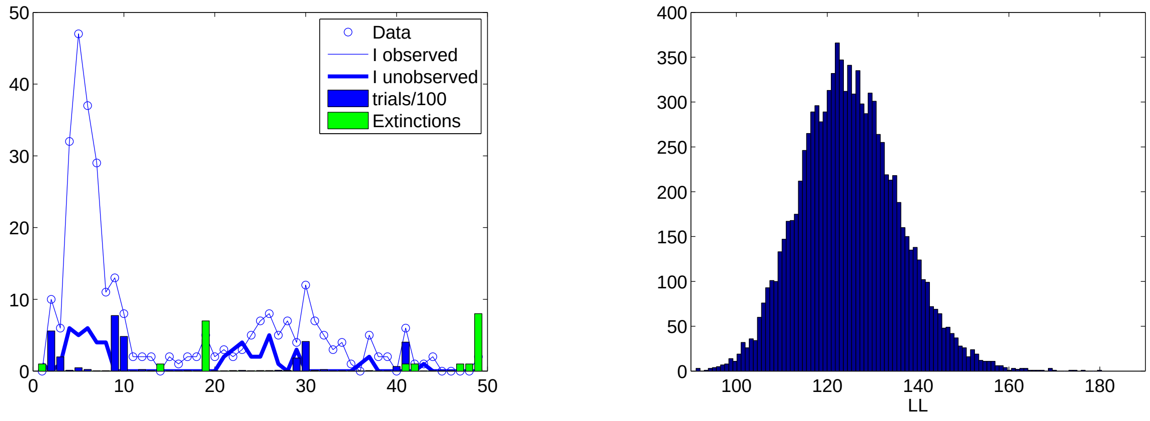

(a) (b)

Figure S9: **a.** An example run of the stochastic model. $I_{observed}$ matches the data (as required). $I_{unobserved}$ shows when the 53 unobserved but symptomatic infections were present. The blue bars show $x_n/100$ for each day. The green bars indicate where extinctions regularly occurred. It is important to note that each run of the model can give different results for timing of extinctions and/or when the algorithm needs to step back one (or more) days. **b.** Histogram of LL for the median parameter values.

If we select a combination of parameters from the tail of the MCMC posterior distribution the stochastic model is far less likely to reproduce the observed data. In fact, if we take the 25th percentile parameter estimates from the Tristan da Cunha posterior distributions, our stochastic model usually fails to find a solution. On the odd occasion that it is successful, LL is significantly worse than is the case for the median parameter estimates. If we take the 75th percentile parameter estimates the stochastic model succeeds about 25% of the time. Again, LL is significantly higher than for the median parameter estimates. Table S1 summarises these results.

Parameter set	Number of runs	Runs completed†	Median LL (interquartile range)
Median	$10,000$	$> 99\%$	124.5 (117.5 – 132.0)
25th percentile	1000	0.90%	153.0 (144.5 – 156.5)
75th percentile	1000	22.4%	163.6 (151.8 – 181.0)

Table S1: Log-likelihoods (LL, as calculated by (S13)) for the outbreak on Tristan da Cunha for the median, 25th percentile ($R_0 = 5.38$, $z = 0.76$, $\alpha = 0.84$, $\rho = 0.45$, $T_w = 11$, $T_e = 1.17$, $T_i = 0.77$) and 75th ($R_0 = 7.60$, $z = 0.92$, $\alpha = 0.96$, $\rho = 0.51$, $T_w = 14$, $T_e = 1.48$, $T_i = 1.27$) percentile parameters. †A run was deemed to have failed to complete if $\sum_{n=1}^{49} x_n > 5 \times 10^5$.

S3.5 Ancillary results from a boarding school in 1918

The applicability of our model to other data-sets was explored with data from an outbreak of influenza at the Saffron Walden boarding school in 1918. 89% of boys living at the school experienced symptomatic infections in a single epidemic wave. Given the high attack rate, the problem of identifiability because of prior immunity as a constraint on epidemic spread is less important. We fitted our model, without waning immunity, to the data. The brevity of the epidemic and lack of multiple attacks makes this a reasonable assumption to make. The data provide no information from which to infer T_w or ϕ.

The results, to be presented in detail elsewhere, support the findings of the main paper. There was little evidence for prior immunity ($z = 0.91$ (0.85 – 1.00)) in this population of school aged children. The inferred symptomatic infection rate ($\alpha = 0.91$ (0.85 – 1.00)) was similar to that for similarly susceptible inhabitants of Tristan da Cunha. The estimated value for R_0 in this setting was 6.90 (5.78 – 8.96), reflecting the ability of influenza virus to spread rapidly in a susceptible population.

S4 Advantages and limitations of our model

The key advantage of our modelling approach is the explicit incorporation of prior immunity and asymptomatic infections as constraints on the observed population incidence of influenza. We were able to evaluate these effects by using detailed whole of population data from the isolated island of Tristan da Cunha, with evidence of re-infection in individuals over a short time period. The RAF data, while less accurate in time (weekly rather than daily collection) and population (likelihood of troop movements within the period) provide complementary conclusions to the Tristan da Cunha experience when evaluated using the dynamic model.

We recognise that our biological assumptions regarding development of immunity are somewhat simplistic and could be improved by allowing for additional complexities. In particular, the immunity arising from any given virus exposure is likely to depend on age of the infected host and prior (lifetime) exposure history.

The population of islanders returned to Tristan da Cunha in 1962, after being evacuated to England when the volcano on the island erupted in 1960. It is entirely plausible that in 1971, the entire population had not been exposed to any form of influenza for some 8-9 years, and was thus susceptible to the H3N2 virus introduced by ship in 1971. In such circumstances we suggest that the relationship between age and susceptibility observed in many other populations would be less evident. In the RAF data-set from a military cohort in 1918, all susceptible hosts were likely to be of similar age with similar histories of exposure to previous infection.

Our model is deterministic and does not allow for heterogeneous mixing. We suggest that the large social gatherings after the arrival of the *Tristania*, to welcome home the four islanders returning from South Africa, would have provided opportunity for rapid viral dissemination, initiating multiple chains of infection, approximating the state of homogeneous mixing. The homogenous mixing assumption is also likely to be an acceptable approximation for conditions within each RAF camp, as personnel were living in close quarters in military barracks. However, it might be argued that there was heterogeneity arising from different timing (asynchrony) of outbreaks in different camps. Such asynchrony would mean that our R_0 estimate (from pooled incidence data) would be less than an estimate based on the data from individual camps, if data were available to make it (Indeed, the latter estimates would be a better reflection of the propensity for influenza to be spread from person to person). Likewise, heterogeneity in the timing of outbreaks in different camps would likely decrease the estimate of z (proportion initially susceptible), and perhaps partially explain the differences between our estimates from RAF camps and from Saffron Walden School in 1918. Further work is in progress to explore these possibilities.

Despite some reservations we believe that the broad-brush conclusions regarding the impact of immunity and asymptomatic infection on spread of influenza presented in the main paper are robust, and provide a reasonable guide to the true state of affairs.

References

[1] A. Gelman, J. B. Carlin, H. S. Stern, and D. B. Rubin. *Bayesian Data Analysis*. Texts in statistical science. Chapman & Hall, London, 2nd edition, 2003. 4
